# Impact of observer experience on multi-detector computed tomography aortic valve morphology assessment and valve size selection for transcatheter aortic valve replacement

**DOI:** 10.1038/s41598-022-23936-w

**Published:** 2022-12-12

**Authors:** Ruben Evertz, Sebastian Hub, Johannes T. Kowallick, Tim Seidler, Bernhard C. Danner, Gerd Hasenfuß, Karl Toischer, Andreas Schuster

**Affiliations:** 1 Department of Cardiology and Pneumology, Georg-August-University Göttingen, University Medical Center Göttingen (UMG), Göttingen, Germany; 2 German Center for Cardiovascular Research (DZHK), Göttingen, Germany; 3 Institute for Diagnostic and Interventional Radiology, Georg-August-University, University Medical Center Göttingen (UMG), Göttingen, Germany; 4 Department of Cardiac, Thoracic and Vascular Surgery, Georg-August-University, University Medical Center Göttingen (UMG), Göttingen, Germany

**Keywords:** Cardiology, Valvular disease

## Abstract

Transcatheter aortic valve replacement (TAVR) has become the standard treatment for aortic stenosis in older patients. It increasingly relies on accurate pre-procedural planning using multidetector computed tomography (MDCT). Since little is known about the required competence levels for MDCT analyses, we comprehensively assessed MDCT TAVR planning reproducibility and accuracy with regard to valve selection in various healthcare workers. 20 randomly selected MDCT of TAVR patients were analyzed using dedicated software by healthcare professionals with varying backgrounds and experience (two structural interventionalists, one imaging specialist, one cardiac surgeon, one general physician, and one medical student). Following the analysis, the most appropriate Edwards SAPIEN 3™ and Medtronic CoreValve valve size was selected. Intra- and inter-observer variability were assessed. The first structural interventionalist was considered as reference standard for inter-observer comparison. Excellent intra- and inter-observer variability was found for the entire group in regard to the MDCT measurements. The best intra-observer agreement and reproducibility were found for the structural interventionalist, while the medical student had the lowest reproducibility. The highest inter-observer agreement was between both structural interventionalists, followed by the imaging specialist. As to valve size selection, the structural interventionalist showed the highest intra-observer reproducibility, independent of the brand of valve used. Compared to the reference structural interventionalist, the second structural interventionalist showed the highest inter-observer agreement for valve size selection [ICC 0.984, 95% CI 0.969–0.991] followed by the cardiac surgeon [ICC 0.947, 95%CI 0.900–0.972]. The lowest inter-observer agreement was found for the medical student [ICC 0.507, 95%CI 0.067–0.739]. While current state-of-the-art MDCT analysis software provides excellent reproducibility for anatomical measurements, the highest levels of confidence in terms of valve size selection were achieved by the performing interventional physicians. This was most likely attributable to observer experience.

## Introduction

Aortic stenosis (AS) is the most common valvular heart disease with increasing prevalence in the elderly population in Europe and North America^1,2^. For its treatment, transcatheter aortic valve replacement (TAVR) has become a well-established option in various patient groups^3–6^. Sufficient pre-procedural anatomical visualisation, quantification of the structures of interest and subsequent adequate valve size selection are all crucial to reduce the incidence of complications such as valve prosthesis dislocation or annulus rupture by an under- or oversized prosthesis^7,8^. Due to its high spatial resolution, multidetector computed tomography (MDCT) has become the standard pre-procedural imaging method in patients undergoing TAVR^9^. Previous studies showed high levels of inter-observer agreement in terms of MDCT measurements and valve size selection, but were primarily focussing on readers with existing imaging experience without experience in structural intervention^10,11^. To date, national guidelines do not define how much imaging experience is required to analyse pre-procedural MDCT scans, or the qualifications necessary to perform the analysis in order to select an appropriate prosthesis size^12^. Therefore, we carried out a comprehensive assessment of MDCT post-processing analyses of reproducibility and accuracy of valve selection in different healthcare professionals including two structural interventionalists, an imaging specialist, a cardiac surgeon, a general physician and a medical student in a tertiary cardiology hospital in order to define minimum post-processing competence levels and provide guidance on required performance for pre-procedural planning.

## Methods

Twenty patients with severe AS as confirmed by echocardiography and who underwent TAVR in 2019 were enrolled; patients with bicuspid AS were excluded. This study was conducted according to the principles of the Helsinki Declaration.

### Multidetector computed tomography

A dual-source CT scanner (SOMATOM Force, Siemens Healthcare GmbH, Erlangen, Germany) was used to generate contrast-enhanced MDCT scans in a prospectively ECG-triggered high-pitch spiral acquisition mode. The region of interest extended from the clavicles to the femoral heads. CT angiography was performed with bolus tracking in the descending aorta using a contrast agent bolus of 80 ml (Imeron 350, Bracco Imaging, Konstanz, Germany) followed by a 40 ml saline chaser, both at a flow rate of 4 ml/sec. Scan parameters were as follows: 2 × 192 × 0.6 mm collimation, 250 ms rotation time, pitch of 3.2, automated tube current adaption. A small field of view data set with medium soft convolution kernel (Siemens Bv36), 0.75 mm slice thickness and 0.5 mm slice increment was generated for the assessment of the aortic annulus, root, and valve morphology and dimensions. All data were analysed using dedicated software (3 Mensio, Structural Heart, V9.1., Pie Medical Imaging, Maastricht, Netherlands).

### Study design

Five different healthcare professionals of varying levels of experience were involved in this study to assess the impact of experience on post-processing of MDCT scans. All observers focused their analysis on the structures which determine valve selection, whereas the vascular approach was not considered. The parameters obtained in this study are displayed in Table 1. The group of observers included two interventional structural heart cardiologists (reference structural interventionalist and a second one), both with more than 5 years of experience in TAVR, one cardiac imaging specialist (cardiologist by training), one cardiac surgeon with more than 5 years of experience in TAVR and SAVR, one general physician and one medical student. All observers were given a brief training on the software provided by the company. This training was comprised of a presentation on how to use the software including an exemplary demonstration of an evaluation in one patient. In addition, all observers underwent a short briefing to standardize the measurements according to the study protocol. All scans were analysed twice in a standardized approach by each observer (with exception of the structural interventionalist two), with at least 4 weeks between the two runs, to assess intra-observer variability in regard to the measured parameters (aortic annulus area, aortic annulus area derived diameter, aortic annulus diameter average, aortic annulus perimeter, sinus of valsalva diameter). The measurements of the structural interventionalists two were used to assess the inter-observer variability in terms of MDCT measurements as well as valve size selection.

**Table 1:** Measured parameters using 3 Mensio for structural heart disease.

MDCT derived measurements of interest		
Aortic annulus	Area	mm^2^
	Area derived diameter	mm
	Diameter average	mm
	Perimeter	mm
Sinus of valsalva	SOV diameter	mm

After each run, each observer had to choose the valve size for the Medtronic CoreValve (23 mm, 26 mm, 29 mm, 34 mm) and the Edwards SAPIEN 3™ valve (23 mm, 26 mm, 29 mm) that were used at the Heart Centre Göttingen, at that time. The selection was based on the measurements and a recommendation sheet provided by the manufacturer (Tables 2 and 3). To determine the inter-observer variability, the first run measurements of the observers were compared to the structural interventionalist, who was considered as reference.

**Table 2:** Size selection sheet for Edwards SAPIEN 3™, image source: “With the kind permission of Edwards Lifesciences”.

Edwards SAPIEN 3™ valve size selection recommendation sheet			
	23 mm Edwards SAPIEN 3™	26 mm Edwards SAPIEN 3™	29 mm Edwards SAPIEN 3™
Native annulus area	338–430 mm^2^	430–546 mm^2^	540–680 mm^2^
Area-derived diameter	20.7–23.4 mm	23.4–26.4 mm	26.2–29.5 mm

**Table 3:** Size selection sheet for Medtronic CoreValve; image source: “With the kind permission of Medtronic GmbH”.

Medtronic CoreValve valve size selection recommendation sheet				
	23 mm Medtronic CoreValve	26 mm Medtronic CoreValve	29 mm Medtronic CoreValve	34 mm Medtronic CoreValve
Annulus diameter	18–20 mm	20–23 mm	23–26 mm	26–30 mm
Annulus perimeter	56.5–62.8 mm	62.8–72.3 mm	72.3–81.7 mm	81.7–94.2 mm
SOV diameter	≥ 25 mm	≥ 27 mm	≥ 29 mm	≥ 31 mm
SOV height	≥ 15 mm	≥ 15 mm	≥ 15 mm	≥ 16 mm

### Statistics

Statistical analysis was conducted using Microsoft Excel 2016 (Microsoft Corporation, Redmond, Washington USA) and IBM SPSS Statistics version 26 for Windows (International Business Machines Corporation (IBM® Corp.), Armonk, New York, USA). Data are expressed as mean ± standard deviations. Normal distribution was tested using the Shapiro–Wilk test. Non-normally distributed data were compared using Mann-Withney U and Kruskal–Wallis tests as appropriate. For between-group comparisons in normally distributed data, t- or ANOVA testing were carried out as appropriate. P-values provided are two-sided, an alpha level of ≤ 0.05 was considered statistically significant.

Furthermore, intra- and inter-observer variability was assessed using three different methods: intraclass correlation coefficients (ICC), Bland Altman analysis, and coefficients of variation (CoV). Bland Altmann analysis reveals “mean differences”. When the compared measurements revealed exactly the same result, all the differences would be equal to zero. A deviation to zero represents the average deviation of measurement x to measurement y^13^. The CoV was defined as the standard deviation of the differences divided by the mean^14,15^. The level of agreement was defined as follows: excellent for ICC > 0.74, good for ICC 0.60–0.74, for ICC 0.40–0.59, and poor for ICC < 0.4^16^.

### Ethical approval

This study was conducted according to the guidelines of the Declaration of Helsinki, and approved by the local ethics committee of the University Medical Center Göttingen (10/5/16). Informed consent was obtained from all subjects involved in the study.

## Results

### Demographics

Patients´ characteristics are displayed in Table 4. A total of 14 (70%) patients were male. Mean age was 78 ± 6 years and ranged from 61 to 90 years. AS was confirmed in all cases by echocardiography with a mean transvalvular peak velocity (V max) of 4.1 ± 0.7 m/s and an average transvalvular mean gradient of 40.3 ± 14.4 mmHg. The estimated mean aortic valve area was 0.7 ± 0.3 cm^2^.

**Table 4:** Patients´ characteristics.

Patients	n = 20
**Demographics**	
Age [years]	78 ± 6 (61–90)
Male	14 (70%)
BMI [kg/m^2^]	24.8 ± 4.1 (16.8–34.2)
**Echocardiographic parameters**	
**Aortic valve**	
V max [m/s]	4.1 ± 0.7 (2.8–5.1)
P mean [mmHg]	40.3 ± 14.4 (17–64)
AVA VTI [cm^2^]	0.7 ± 0.3 (0.2–1.0)
SVI [ml/m^2^]	35.0 ± 9.5 (9.5–59.4)
**Left ventricle**	
LVEF [%]	49.3 ± 9.4 (25–55)
LVEDD [mm]	46.2 ± 8.6 (32–68)
**Comorbidities**	
HT	19 (95%)
AF	14 (70%)
DM	4 (20%)
CAD	13 (65%)
COPD	2 (10%)

BMI: Body-Mass-Index; V max: transvalvular peak velocity; P mean: transvalvular mean gradient; AVA VTI: aortic valve area measured by velocity time integral; SVI: stroke volume index; LVEF: left ventricular ejection fraction; LVEDD: left ventricular end diastolic diameter; HT: hypertension; AF: atrial fibrillation; DM: diabetes mellitus; CAD: coronary artery disease; COPD: chronic obstructive pulmonary disease.

The most common comorbidity was hypertension (95%), followed by atrial fibrillation and coronary artery disease (70% and 65%, respectively). Diabetes mellitus was present in 4 patients (20%) and chronic obstructive pulmonary disease in 2 patients (10%).

### Assessment of aortic anatomy for valve size selection

**Figure 1:**
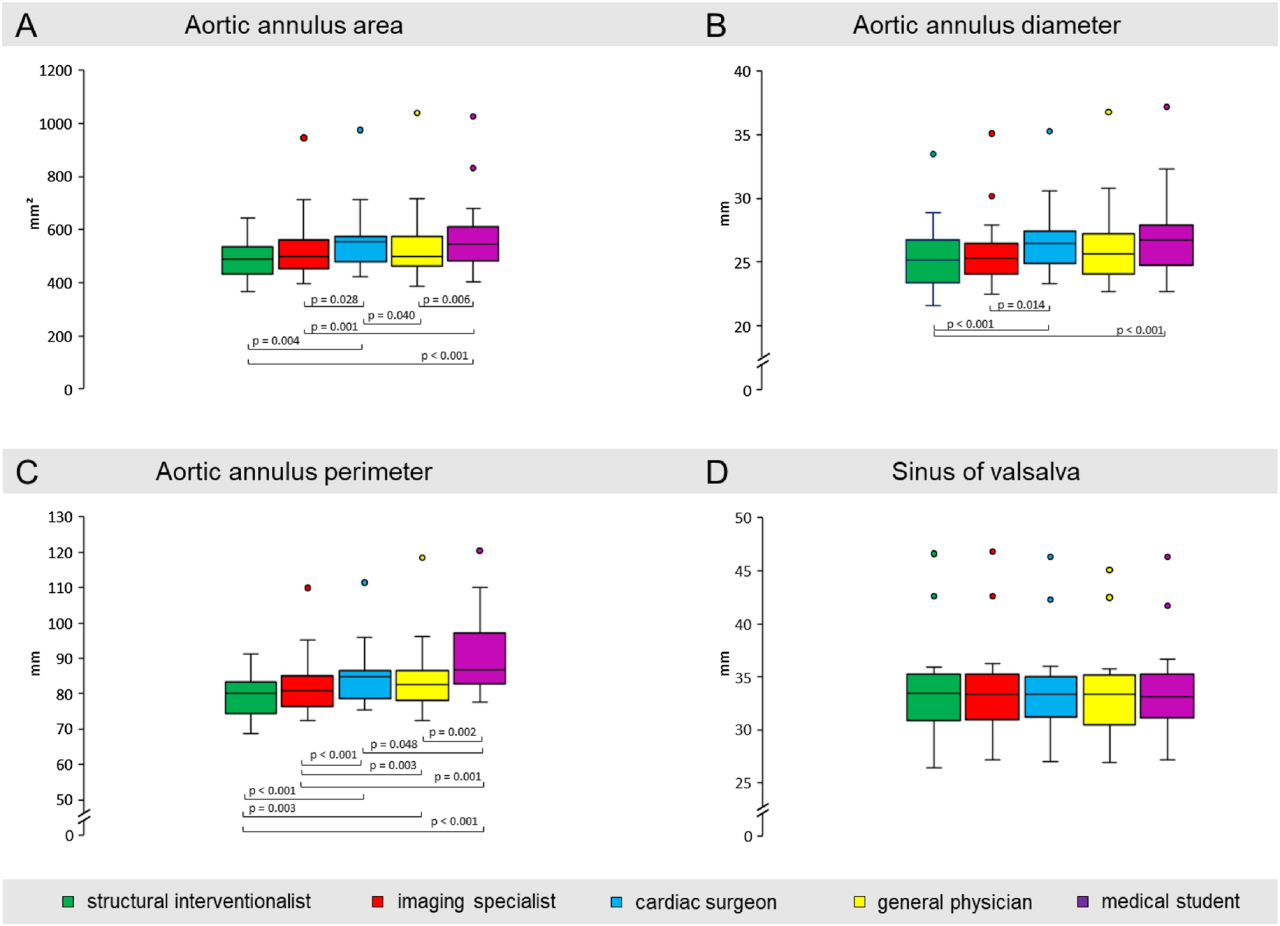
Inter-observer measurement presentations of (**A**) Aortic annulus area; (**B**) Aortic annulus average diameter; (**C**) Aortic annulus perimeter; (**D**) Sinus of Valsalva; *p*-values < 0.05 indicate significant differences.

Mean annulus area measured by the different observers ranged between 502.87 mm^2^ and 571.32 mm^2^, which resulted in significant differences between the observers (*p* < 0.001). The mean aortic annulus area derived diameter was measured between 25.1 ± 2.9 mm and 26.8 ± 3.3 mm and differed significantly between the observers (p < 0.001). The aortic annulus area derived annulus diameter differed numerically only minimally from the measured averaged aortic annulus diameter and ranged between 25.4 ± 2.7 mm and 27.0 ± 3.4 mm. Mean annulus perimeter varied from 80.3 ± 9.1 mm to 91.0 ± 11.9 mm with significant differences between the observers (*p* < 0.001). The average measured diameter of the sinus of valsalva (SOV) ranged between 33.3 ± 4.5 mm and 33.6 ± 4.9 mm (*p* = 0.504). Figure 1A–D illustrates the measured values by the 5 observers and also indicates significant inter-observer differences for all aforementioned parameters (online data supplement Figure S1 includes also structural interventionalist two).

### Valve size selection

Overall, for the Edwards SAPIEN 3™ valve the medium-sized valve (26 mm) was chosen most frequently (49%) followed by the largest one (29 mm, 38%) and the smallest one (23 mm, 13%). With regard to the Medtronic CoreValve, the most frequently selected valve size was the largest (34 mm, 52%) followed by the medium (29 mm, 40%) and small (26 mm, 8%) sizes. The smallest Medtronic CoreValve (23 mm) was not selected by any of the operators. There was no difference on an individual observer level. Details are given in the online data supplement (online data supplement Table S1–S2).

### Intra- and inter-observer variability of CT measurements

Excellent intra-observer agreement was seen for all observers (Table 5 and online data supplement Figure S2–S6). The structural interventionalist showed the best intra-observer agreement and reproducibility for all measured MDCT parameters, with the exception of SOV. The medical student had the lowest reproducibility in 3 out of 5 categories (aortic annulus area derived diameter, aortic annulus average diameter and aortic annulus perimeter). Numerically small but statistically significant differences were observed between the initial and the repeated analysis runs for the following parameters:

**Table 5:** Intra-observer agreement and reproducibility.

	Structural interventionalist			Imaging specialist			Cardiac surgeon		
	Mean Difference (SD)	ICC (95%CI)	COV (%)	Mean Difference (SD)	ICC (95%CI)	COV (%)	Mean Difference (SD)	ICC (95%CI)	COV (%)
**Intra-observer**									
Area [mm^2^]	0.57 (7.63)	0.999 (0.998–1.000)	1.52	− 6.45 (35.45)	0.980 (0.950–0.992)	6.7	27.02 (33.27)	0.973 (0.856–0.992)	6.13
Area derived diameter [mm]	0.00 (0.21)	0.999 (0.997–1.000)	0.83	− 0.14 (0.89)	0.976 (0.939–0.990)	3.45	0.68 (0.83)	0.966 (0.815–0.989)	3.16
Avg. diameter [mm]	0.12 (0.41)	0.994 (0.986–0.998)	1.62	− 0.30 (1.03)	0.967 (0.917–0.987)	3.95	0.69 (0.71)	0.972 (0.770–0.992)	2.68
Perimeter [mm]	-0.01 (0.69)	0.999 (0.997–0.999)	0.86	− 0.48 (3.07)	0.970 (0.926–0.988)	3.73	2.1 (2.81)	0.962 (0.829–0.988)	3.36
SOV average diameter [mm]	-0.21 (0.62)	0.996 (0.989–0.998)	1.85	− 0.04 (0.42)	0.998 (0.995–0.999)	1.24	0.07 (0.60)	0.996 (0.989–0.998)	1.79

	General physician			Medical student					
	Mean Difference (SD)	ICC (95% CI)	COV (%)	Mean Difference (SD)	ICC (95%CI)	COV (%)			
**Intra-observer**									
Area [mm^2^]	− 11.52 (71.95)	0.935 (0.837–0.974)	13.54	− 3.41 (61.39)	0.958 (0.893–0.983)	10.71			
Area derived diameter [mm]	− 0.06 (0.81)	0.983 (0.957–0.993)	3.13	− 0.07 (1.46)	0.949 (0.870–0.980)	5.45			
Average diameter [mm^2^]	0.14 (0.96)	0.976 (0.940–0.991)	3.66	0.02 (1.34)	0.957 (0.891–0.983)	4.99			
Perimeter [mm^2^]	0.66 (2.93)	0.997 (0.944–0.991)	3.51	4.33 (8.00)	0.823 (0.518–0.932)	9.01			
SOV average diameter [mm]	0.23 (0.98)	0.988 (0.971–0.995)	2.96	0.12 (0.89)	0.990 (0.976–0.996)	2.65			

Results are reported as mean (SD). SOV: sinus of valsalva; ICC: intraclass-correlation coefficient; CoV: coefficient of variation; SD: standard deviation; CI: confidence interval.

1. Annulus area, aortic annulus area derived diameter, aortic annulus average diameter and aortic annulus perimeter in the measurements by the cardiac surgeon; 2. The aortic annulus perimeter in the measurements of the medical student; 3. The SOV measurements of the general physician (online data supplement Figures S7–S10).

There were no statistically significant differences between the two runs for the structural interventionalist and the imaging specialist.

Compared to the structural interventionalist the inter-observer agreement and reproducibility were excellent for all analysed data including the analyses of the structural interventionalist two, the imaging specialist, the cardiac surgeon and the general physician. In addition, the medical student showed also excellent reproducibility for 4 out of 5 recorded parameters. Only the aortic annulus perimeter agreement did not reach excellent inter-observer reproducibility and was considered “good” compared to the structural interventionalist. The highest agreement with the structural interventionalist was observed for the second structural interventionalist followed by the imaging specialist, the general physician and the cardiac surgeon (Table 6).

**Table 6:** Inter-observer agreement and reproducibility.

	Structural interventionalist–structural interventionalist two			Structural interventionalist–imaging specialist			Structural interventionalist–cardiac surgeon		
	Mean Difference (SD)	ICC (95%CI)	COV (%)	Mean Difference (SD)	ICC (95%CI)	COV (%)	Mean Difference (SD)	ICC (95%CI)	COV (%)
**Inter-observer**									
Area [mm^2^]	− 2.36 (23.4)	0.991 (0.977–0.996)	4.65	− 23.14 (34.20)	0.973 (0.893–0.991)	6.65	− 53.50 (36.33)	0.934 (0.112–0.984)	6.86
Area derived diameter [mm]	− 0.07 (0.60)	0.989 (0.972–0.996)	2.38	− 0.61 (0.87)	0.966 (0.859–0.988)	3.43	− 1.34 (0.95)	0.917 (0.084–0.980)	3.69
Avg. diameter [mm]	− 0.02 (0.69)	0.984 (0.960–0.994)	2.72	− 0.45 (0.91)	0.967 (0.907–0.988)	3.57	− 1.31 (0.95)	0.917 (0.084–0.980)	3.59
Perimeter [mm]	− 0.57 (2.01)	0.987 (0.968–0.995)	2.49	− 1.87 (2.54)	0.969 (0.863–0.990)	3.13	− 4.54 (3.11)	0.904 (0.005–0.977)	3.77
SOV average diameter [mm]	− 0.03 (0.61)	0.996 (0.990–0.998)	1.82	− 0.13 (0.51)	0.997 (0.992–0.999)	1.53	− 0.01 (0.56)	0.997 (0.991–0.999)	1.67

	Structural interventionalist–general physician			Structural interventionalist–medical student					
	Mean Difference (SD)	ICC (95%CI)	COV (%)	Mean Difference (SD)	ICC (95%CI)	COV (%)			
**Intra-observer**									
Area [mm^2^]	− 22.91 (47.15)	0.965 (0.902–0.987)	9.17	− 68.45 (54.62)	0.901 (0.136–0.974)	10.14			
Area derived diameter [mm]	− 0.81 (0.92)	0.960 (0.752–0.988)	3.60	− 1.66 (1.25)	0.891 (0.048–0.972)	4.82			
Average diameter [mm]	− 0.8 (1.02)	0.953 (0.773–0.985)	3.97	− 1.56 (1.26)	0.897 (0.137–0.973)	4.83			
Perimeter [mm]	− 3.54 (2.95)	0.944 (0.398–0.986)	3.60	− 10.72 (6.30)	0.708 (0.215–0.920)	7.35			
SOV average diameter [mm]	0.24 (1.10)	0.986 (0.965–0.994)	3.31	− 0.04 (0.74)	0.994 (0.985–0.998)	2.20			

Results are reported as mean (SD). SOV: sinus of valsalva; ICC: intraclass-correlation coefficient; CoV: coefficient of variation; SD: standard deviation; CI: confidence interval.

### Intra- and inter-observer variability for valve size selection

All observers reached an excellent level of intra-observer agreement for valve size selection. When estimating the ICC without taking into account the different valve manufacturers, excellent intra-observer agreements were found for all observers, with the highest intra-observer agreement for the structural interventionalist [ICC 0.991, 95% CI 0.983–0.995], followed by the cardiac surgeon [ICC 0.944, 95% CI 0.893–0.970] the general physician [ICC 965, 95% CI 0.934–0.982], the imaging specialist [ICC 0.909, 95% CI 0.827–0.952] and the medical student [ICC 0.910, 95% CI 0.830–0.952]. When assessing in terms of manufacturers, the ICC for selection of the Edwards SAPIEN 3™ valve ranged between 0.776 and 0.945, whereas ICC for the Medtronic CoreValve varied between 0.816 and 0.989. The structural interventionalist had the best reproducibility for both valve types (Edwards SAPIEN 3™: ICC 0.945, 95% CI 0.826–0.978, Medtronic CoreValve: ICC 0.989, 95% CI 0.973–0.996). A detailed overview of the intra-observer ICC is shown in Table 7.

**Table 7:** Intra- and inter-observer ICC for valve selection.

Intra-observer	Edwards SAPIEN 3™		Medtronic CoreValve	
	ICC	95% CI	ICC	95% CI
Structural interventionalist	0.945	(0.826–0.978)	0.989	(0.973–0.996)
Imaging specialist	0.818	(0.547–0.927)	0.816	(0.532–0.928)
Cardiac surgeon	0.845	(0.611–0.938)	0.897	(0.744–0.959)
General physician	0.897	(0.744–0.959)	0.954	(0.884–0.982)
Medical student	0.776	(0.425–0.912)	0.856	(0.633–0.943)

Inter-observer	Valve type			
	Edwards SAPIEN 3™		Medtronic CoreValve	
	ICC	95% CI	ICC	95% CI
Structural interventionalist – Structural interventionalist 2	0.977	(0.941–991)	0.966	(0.915–0.987)
Structural interventionalist – Imaging specialist	0.831	(0.485–0.938)	0.867	(0.670–0.947)
Structural interventionalist – Cardiac surgeon	0.871	(0.626–0.951)	0.880	(0.678–0.953)
Structural interventionalist – General physician	0.855	(0.585–0.945)	0.889	(0.722–0.956)
Structural interventionalist – Medical student	0.737	(0.092–0.909)	0.897	(0.723–0.960)

ICC: intraclass-correlation coefficient; CI: confidence interval.

**Figure 2:**
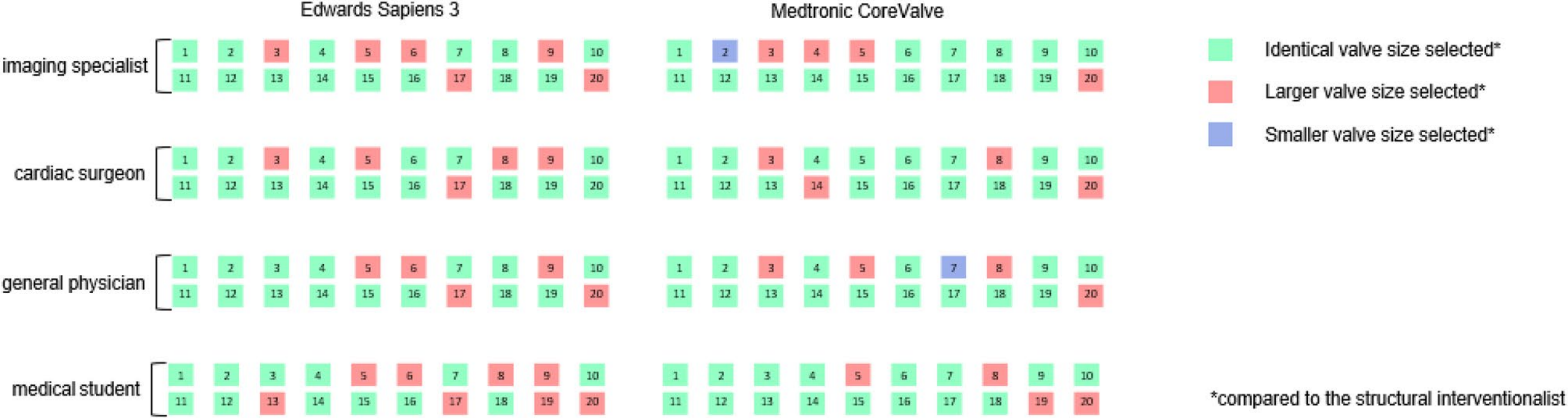
Valve size selection compared to the interventionalist for each patient, separated by valve type.

In terms of the inter-observer agreement excellent agreement was found for the structural interventionalist two [ICC 0.984, 95% CI 0.969–0.991], the cardiac surgeon [ICC 0.947, 95% CI 0.900–0.972], the general physician [ICC 0.944, 95% CI 0.895–0.971] and the imaging specialist [ICC 0.933, 95% CI 0.873–0.964] while the medical student only reached a fair agreement [ICC 0.507, 95% CI 0.067–0.739] compared to the reference standard (structural interventionalist). When assessing according to the different valve manufacturers, the highest level of agreement was found for the structural interventionalist two (Edwards SAPIEN 3™: ICC 0.977, 95% CI 0.941–0.991; Medtronic CoreValve: ICC 0.966, 95% CI 0.915–0.987) followed by the cardiac surgeon with an ICC of 0.871 [95% CI 0.626–0.951) for the Edwards SAPIEN 3™ valve. The lowest agreement was found for the medical student [ICC 0.737, 95% CI 0.092–0.909). In regard to the Medtronic valve size selection, there was excellent agreement at comparable levels for all observers, with the highest for the medical student [ICC 0.897, 95% CI 0.723–0.960] and the lowest for the imaging specialist [ICC 0.867, 95% CI 0.670–0.947). A detailed representation of the ICC is given in Table 7. Furthermore, Fig. 2 depicts the valve size selection agreement of the different observers and the structural interventionalist, whereas Figure S11 of the online data supplement illustrates the annulus area measurements in three selected patients where the measurements resulted in different valve size selections as compared to the structural interventionalist.

## Discussion

To our knowledge, this is the first study investigating the impact of the experience of different healthcare professionals involved in the field of MDCT measurements and valve size selection prior to TAVR. We included several different healthcare professionals: two structural interventionalists, a cardiac surgeon, an imaging specialist, a general physician and a medical student. The following findings are noteworthy: First, MDCT TAVR planning analyses were feasible and robust, with minimal differences between all observers studied, after they all received a brief introduction to the analysis software. Second, among the different observers (excluding the second structural interventionalist) the imaging specialist had the highest and the medical student the lowest agreement with the reference structural interventionalist. Third, in terms of valve size selection the structural interventionalist had the highest level of consistency between repeated analyses which was superior to all other observers. This suggests that besides an adequate analysis of pre- TAVR MDCT scans, clinical and interventional experience should be a prerequisite for consistent and safe procedural planning. Taken together, high quality measurements of the relevant aortic anatomy can be obtained relatively easily based on state-of-the-art MDCT TAVR planning scans, which can then be utilized for accurate determination of the intervention.

It is known that with increasing experience—defined by the number of TAVR procedures performed—the rate of periprocedural complications (mortality, vascular complications, bleeding) decreases^17^. An accurate selection of the valve size also contributes to a reduced complication rate. Therefore, accurate pre-procedural imaging is crucial to assure optimal patient outcome. Transoesophageal echocardiography was initially used to estimate the right valve size, but nowadays MDCT has become the standard procedure, which has led to a reduction in paravalvular regurgitation^18,19^. In line with previous MDCT studies, our study demonstrates excellent intra-observer and inter-observer results for all observers involved, independent of the level of experience, when following a standardized analysis procedure^10,11,19–23^. Due to the high measurement accuracy achieved with MDCT, it initially seems counterintuitive that there were differences in the selection of the valve sizes between and within the observers in the current study. One possible explanation may be that the structural interventionalists traced the border of the region of interest consistently within the outer contrast region, while the other observers delineated the borders around the contrast region resulting in slightly smaller measurements of the interventionalists (please see Figure S11). In cases with a high AVC load the interventionalists bisected the calcifications considering the blooming artefact, which was not as consistently performed by the other observers. Both may have impacted the measurements slightly and consequently valve size selection. A further possible explanation is, that valve size selection is not based on one single parameter but rather, when using the recommendation sheet, on up to four different parameters. There is a slight overlap where one or the other valve size may be chosen (Tables 2 and 3). Therefore, differences may be explained by cases in which the different parameters do not fit one size only, or in the presence of measurements between 2 valve sizes. Horehledova et al. showed, that only 30 to 60% of annulus diameter, annulus area and annulus perimeter measurements indicated the same prosthesis size^24^. In such cases, the level of clinical experience observer becomes more relevant. This may be why the best intra-observer agreement regarding valve size selection was observed for the structural interventionalist, who encounters similar scenarios in their clinical routine and thus targets them in a more structured manner. This may also be the explanation why the structural interventionalist always selected the same valve size in cases of borderline levels of the sizing parameters, while the other observers were not as consistent in this aspect. In addition, the level of experience of the observer most likely also plays a role in the measurements: a higher agreement was again seen in our study for the structural interventionalist compared to the other observers. While non-interventionalists probably relied mostly on their measured parameters and selected the valve size accordingly, the structural interventionalists may have also intuitively based their decisions on appearance and anatomy, e.g. calcium distribution. However, to reduce peri-interventional complications during TAVR, aspects additional to the dimensional measurements (Table 1) have to be considered. These include the shape and size of the aortic annulus, the LVOT ascending aorta angle as well as the extent and distribution of aortic valve calcification. A comprehensive assessment of all parameters involved make it possible to reduce the risk of paravalvular regurgitation, which is associated with worse outcome^25^. Therefore, it is not surprising that we observed differences in valve size selection solely based on MDCT aortic annulus measurements by the observers who were not specialized in structural interventions. The same is true for reducing the risk of a rare but life-threatening aortic annulus rupture. The risk of annulus rupture is associated with high amounts of LVOT calcification and also with prosthetic valve oversizing^26^. All these aspects are not represented in the valve size selection recommendation sheet; they are solely based on clinical experience. There is a multitude of evidence that dedicated training can further improve operator performance^27–29^. Therefore respective programs should be defined and incorporated in relevant guidelines. Nevertheless the excellent intra-observer reproducibility of the aforementioned parameters for all observers is quite reassuring in that these clinical decisions were based on very robust data that can be accurately and safely assessed and then interpreted by an experienced physician.

The software for structural heart disease used in the current study offers an excellent intra- and inter-observer reproducibility in regard to aortic valve measurements. This is likely due to its simplicity and the usability of the program as well as the good image quality of the MDCT technology, which, in contrast to echocardiography, is independent of the examiner. Taken together, successful pre-TAVR MDCT imaging should always be analysed by the actual implanting physician with subsequent valve size selection to allow for safe planning of the procedure based on individual anatomical and clinical patient data. In this regard, our study suggests that clinical experience is likely more important for adequate decision making as compared to the MDCT measurements which can be very accurately performed with generation of highly reproducible measurements after a brief introduction to current MDCT software programs.

Furthermore, borderline decisions on valve size selection will never fully rely on standardized measurement recommendation sheets but rather on the observer’s experience. In clinical practice steps for appropriate selection of a device in borderline cases should always include: 1. a repeated analysis of the annulus area and annulus perimeter to rule out measurement errors; 2. the amount and distribution of calcification and the height of the coronary arteries should be taken into consideration; 3. in situations with large calcifications in the device landing zone ,the smaller valve should be chosen to reduce the risk of an annulus rupture; 4. if there is only a small amount of calcification, the larger valve size should be considered because of a resulting larger valve area and a lower risk of a paravalvular regurgitation; 5. if possible, in borderline situations a second experienced structural interventionalist should be involved with subsequent selection of the adequate valve size in a team approach.

## Limitations

Some limitations of our study need to be addressed. First, our results are derived from a single vendor software package and may not apply to different analyses tools. However, the software package used is well established in clinical routine, and thus our results can be considered clinically meaningful. Second, the selection of the valve size was only based on the measurements of the annulus and the SOV and did not take the vascular access or coronary anatomy into account. However, since this was true for all observers, this bias can be considered a systematic bias. Third, each group of “differently experienced healthcare professionals” (aside from the structural interventionlists) were represented by one person only, all of whom had varying exposure to MDCT measurements before the study was started. Finally, our study included Medtronic CoreValve and the Edwards SAPIEN 3™ valves which were standard devices at the time of data collection.

## Conclusions

MDCT planning and accurate valve size selection remains essential for TAVR without complications. Current state-of-the-art MDCT analysis software provides excellent reproducibility for anatomical measurements irrespective of the level of pre-existing experience. However, the highest levels of confidence in terms of valve size selection are achieved by implanting physicians, which can likely be attributed to observer experience, reflecting current clinical practice.

## Supplementary Information

Supplementary Information.

## Data Availability

All data that support the findings of this study are available upon request from the corresponding author.
