# Supplementary material for: Impact of observer experience on multi-detector computed tomography aortic valve morphology assessment and valve size selection for transcatheter aortic valve replacement
Source: Sci Rep. 2022 Dec 12;12:21430. doi: 10.1038/s41598-022-23936-w (PMC9744877; doi:10.1038/s41598-022-23936-w)
Supplement: Supplementary file 1 — Supplementary Information. [file 41598_2022_23936_MOESM1_ESM.docx]

**Supplement**

**Impact of observer experience on multi-detector computed tomography aortic valve morphology assessment and valve size selection for transcatheter aortic valve replacement**

Ruben Evertz^1,2^, Sebastian Hub^1,2^, Johannes T. Kowallick^2,3^, Tim Seidler^1,2^, Bernhard C. Danner^2,4^, Gerd Hasenfuß^1,2^, Karl Toischer^1,2^, Andreas Schuster^1,2^

1 Department of Cardiology and Pneumology, University Medical Center Göttingen (UMG), Göttingen, Germany

2 German Center for Cardiovascular Research (DZHK), Göttingen, Germany

3 Institute for Diagnostic and Interventional Radiology, University Medical Center Göttingen (UMG), Göttingen, Germany

4 Department of Thoracic and Cardiovascular Surgery, University Medical Center Göttingen (UMG), Göttingen, Germany

Correspondence:

Andreas Schuster, MD, PhD, MBA

University Medical Centre

Georg-August-University Göttingen

Department of Cardiology and Pneumology

Robert-Koch-Str. 40

37099 Göttingen, Germany

Tel.: +49 551 39 20870

Fax.: +49 551 39 22026

E-Mail: [andreas_schuster@gmx.net](mailto:andreas_schuster@gmx.net)

|  | Edwards SAPIEN 3^TM^  23 mm | Edwards SAPIEN 3^TM^  26 mm | Edwards SAPIEN 3^TM^  29 mm |
| --- | --- | --- | --- |
| Structural interventionalist | 5 (25 %) | 10 (50 %) | 5 (25 %) |
| Structural interventionalist two | 6 (30 %) | 9 (45 %) | 5 (25 %) |
| Imaging specialist | 2 (10 %) | 10 (50 %) | 8 (40 %) |
| Cardiac surgeon | 3 (15 %) | 9 (45 %) | 8 (40 %) |
| General physician | 2 (10 %) | 11 (55 %) | 7 (35 %) |
| Medical student | 1 (5 %) | 9 (45 %) | 10 (50 %) |
| Total without structural interventionalist two | 13 (13 %) | 49 (49 %) | 38 (38 %) |
| Total including structural interventionalist two | 19 (15.8) | 58 (48.3) | 43 (35.8) |

**Table S1:** Frequency of different Edwards SAPIEN 3^TM^ valve size selection separated by the different observer and in total expressed as absolute number and percentage.

|  | Medtronic CoreValve  23 mm | Medtronic CoreValve  26 mm | Medtronic CoreValve  29 mm | Medtronic CoreValve  34 mm |
| --- | --- | --- | --- | --- |
| Structural interventionalist | 0 (0 %) | 3 (15 %) | 8 (40 %) | 9 (45 %) |
| Structural interventionalist two | 0 (0 %) | 3 (15%) | 9 (45 %) | 8 (40%) |
| Imaging specialist | 0 (0 %) | 0 (0 %) | 11 (55 %) | 9 (45 %) |
| Cardiac surgeon | 0 (0 %) | 2 (10 %) | 6 (30 %) | 12 (60 %) |
| General physician | 0 (0 %) | 2 (10 %) | 7 (35 %) | 11 (55 %) |
| Medical student | 0 (0 %) | 1 (5 %) | 8 (40 %) | 11 (55 %) |
| Total without structural interventionalist two | 0 (0 %) | 8 (8 %) | 40 (40 %) | 52 (52 %) |
| Total including structural interventionalist two | 0 (0 %) | 11 (9 %) | 49 (41 %) | 60 (50 %) |

**Table S2:** Frequency of different Medtronic CoreValve valve size selection separated by the different observer and in total expressed as absolute number and percentage.


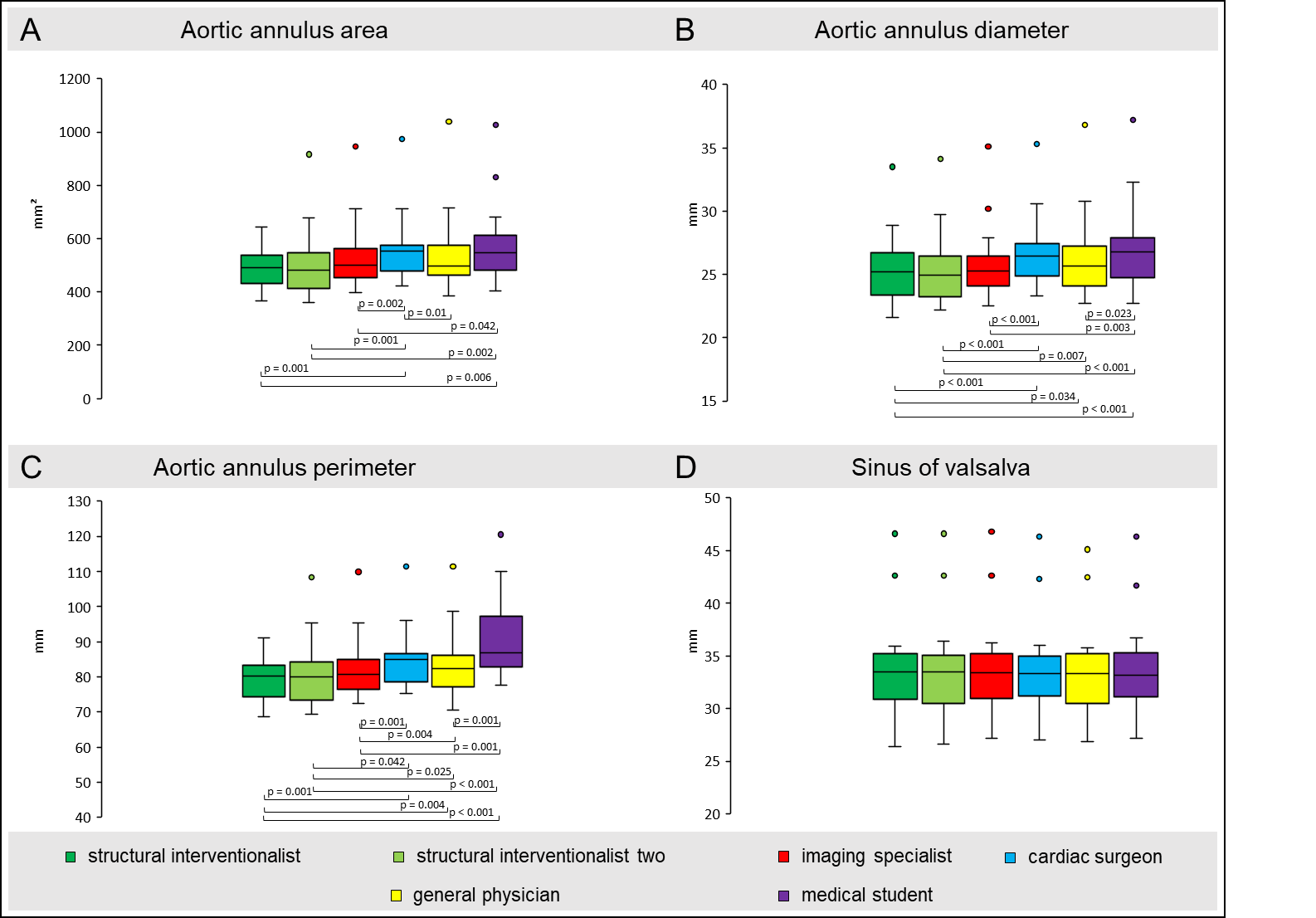


**Figure S1:** Inter-observer measurement presentations of A) Aortic annulus area; B) Aortic annulus average diameter; C) Aortic annulus perimeter; D) Sinus of Valsalva; p-values <0.05 indicate significant differences.

Intra-Observer

structural interventionalist

imaging specialist

cardiac surgeon

Intra-observer difference for Annulus area [mm²]

Intra-observer difference for Annulus area [mm²]

Intra-observer difference for Annulus area [mm²]

Annulus area [mm²]

Annulus area [mm²]

Annulus area [mm²]

Intra-observer difference for Annulus area [mm²]

Intra-observer difference for Annulus area [mm²]

Annulus area [mm²]

Annulus area [mm²]

Intra-Observer

general physician

medical student

**Figure S2** Intra-observer agreement of Annulus area. Bland-Altmann plots with limits of agreement (95% confidence intervals) demonstrate reproducibility of MDCT derived Annulus diameter. MDCT: multidetector computed tomography.

Intra-Observer

structural interventionalist

imaging specialist

cardiac surgeon

Intra-observer difference for Area derived Annulus diameter [mm]

Intra-observer difference for Area derived Annulus diameter [mm]

Intra-observer difference for Area derived Annulus diameter [mm]

Area derived Annulus diameter [mm]

Area derived Annulus diameter [mm]

Area derived Annulus diameter [mm]

Intra-observer difference for Area derived Annulus diameter [mm]

Intra-observer difference for Area derived Annulus diameter [mm]

Area derived Annulus diameter [mm]

Area derived Annulus diameter [mm]

Intra-Observer

general physician

medical student

**Figure S3** Intra-observer agreement of Area derived Annulus diameter. Bland-Altmann plots with limits of agreement (95% confidence intervals) demonstrate reproducibility of MDCT derived Annulus diameter. MDCT: multidetector computed tomography.

Intra-Observer

structural interventionalist

imaging specialist

cardiac surgeon

Intra-observer difference for Annulus diameter [mm]

Intra-observer difference for Annulus diameter [mm]

Intra-observer difference for Annulus diameter [mm]

Annulus diameter [mm]

Annulus diameter [mm]

Annulus diameter [mm]

Intra-observer difference for Annulus diameter [mm]

Intra-observer difference for Annulus diameter [mm]

Annulus diameter [mm]

Annulus diameter [mm]

Intra-Observer

general physician

medical student

**Figure S4** Intra-observer agreement of Annulus diameter. Bland-Altmann plots with limits of agreement (95% confidence intervals) demonstrate reproducibility of MDCT derived Annulus diameter. MDCT: multidetector computed tomography.

Intra-Observer

structural interventionalist

imaging specialist

cardiac surgeon

Intra-observer difference for Annulus perimeter [mm]

Intra-observer difference for Annulus perimeter [mm]

Intra-observer difference for Annulus perimeter [mm]

Annulus perimeter [mm]

Annulus perimeter [mm]

Annulus perimeter [mm]

Intra-observer difference for Annulus perimeter [mm]

Intra-observer difference for Annulus perimeter [mm]

Annulus perimeter [mm]

Annulus perimeter [mm]

Intra-Observer

general physician

medical student

**Figure S5** Intra-observer agreement of Annulus perimeter. Bland-Altmann plots with limits of agreement (95% confidence intervals) demonstrate reproducibility of MDCT derived Annulus diameter. MDCT: multidetector computed tomography.

Intra-Observer

structural interventionalist

imaging specialist

cardiac surgeon

Intra-observer difference for averaged SOV [mm]

Intra-observer difference for averaged SOV [mm]

Intra-observer difference for averaged SOV [mm]

Averaged SOV [mm]

Averaged SOV [mm]

Averaged SOV [mm]

Intra-observer difference for averaged SOV [mm]

Intra-observer difference for averaged SOV [mm]

Averaged SOV [mm]

Averaged SOV [mm]

Intra-Observer

general physician

medical student

**Figure S6** Intra-observer agreement of averaged SOV Diameter. Bland-Altmann plots with limits of agreement (95% confidence intervals) demonstrate reproducibility of MDCT derived Annulus diameter. MDCT: multidetector computed tomography; SOV: sinus of Valsalva.


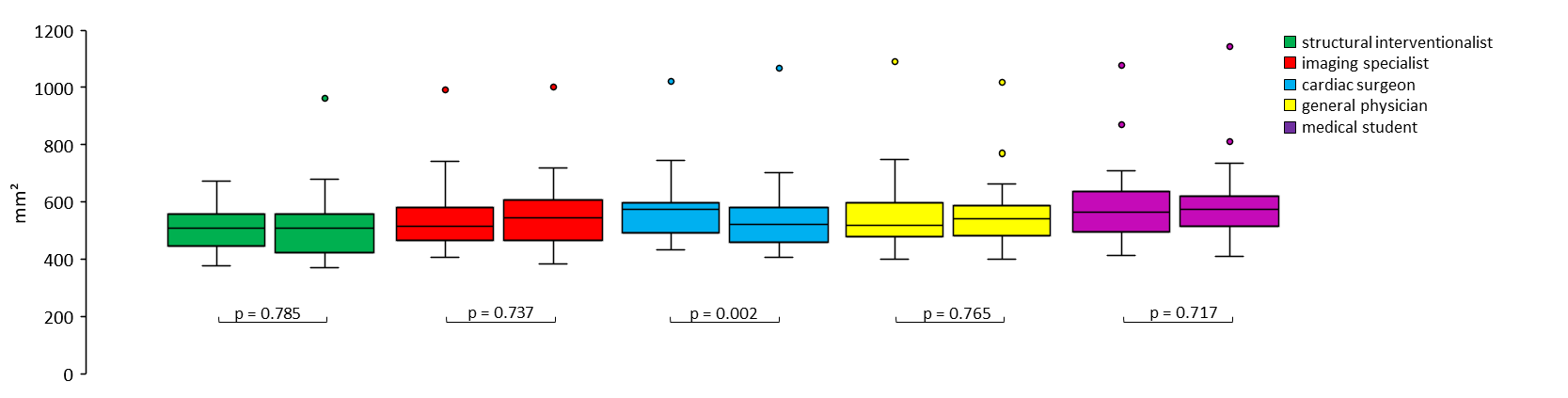


**Figure S7** Illustration of the intra-observer reproducibility for Annulus area measurements.


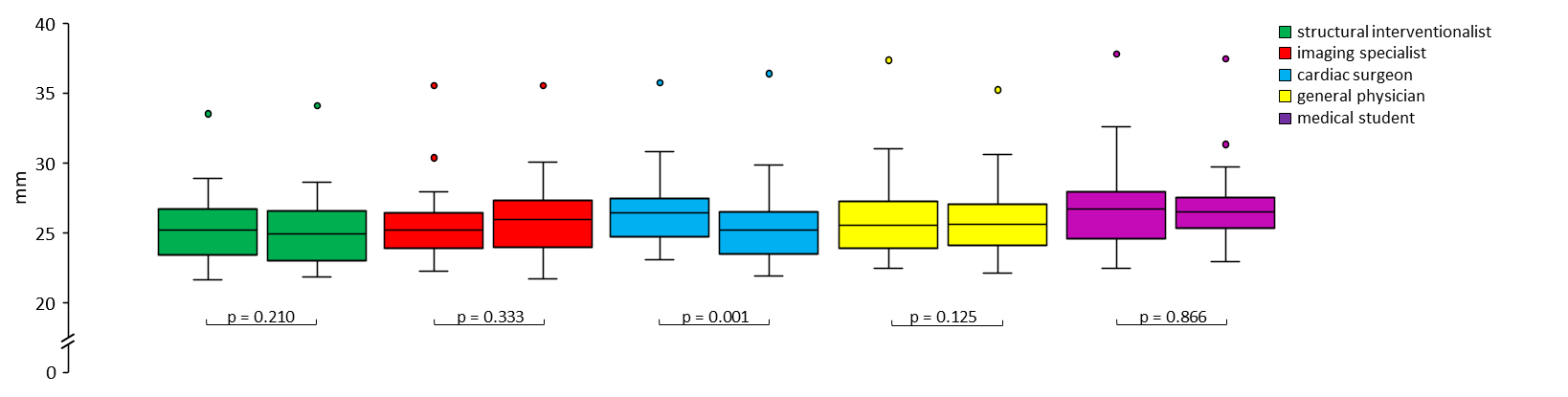


**Figure S8** Illustration of the intra-observer reproducibility for averaged Annulus diameter measurements.


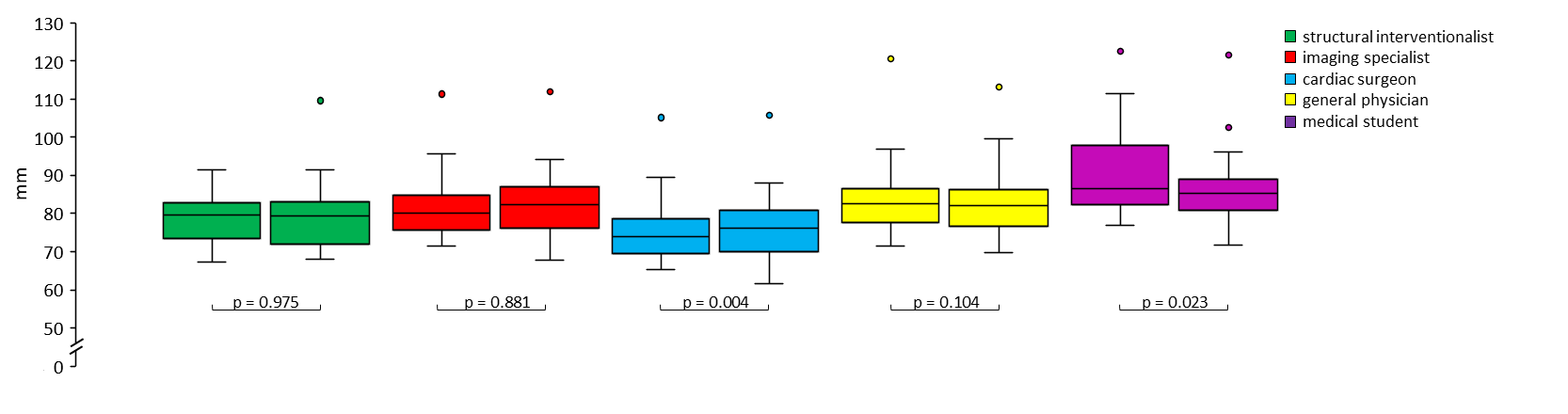
**Figure S9** Illustration of the intra-observer reproducibility for Annulus perimeter measurements.


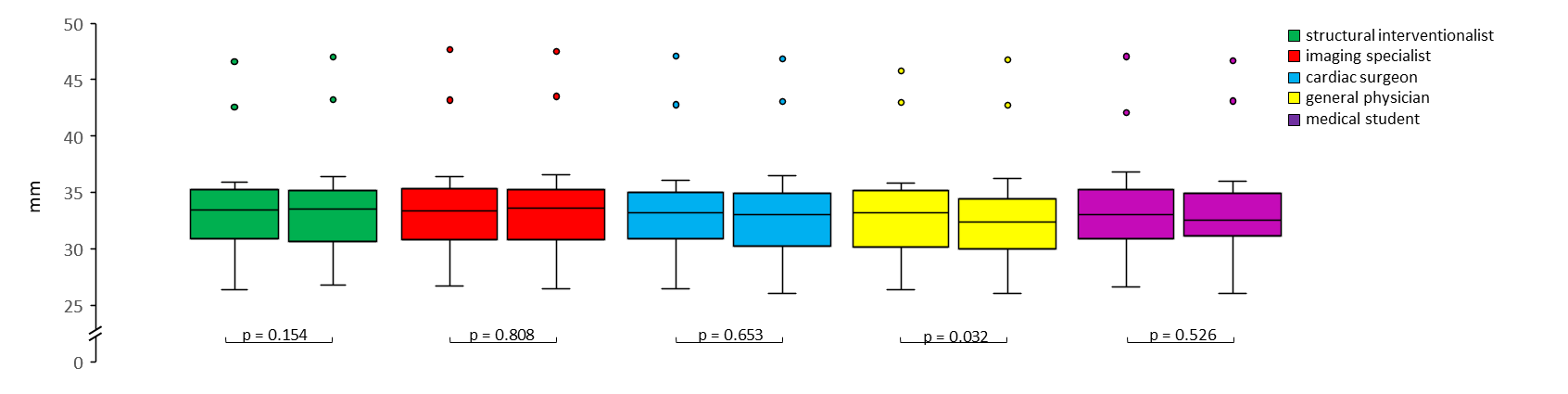
**Figure S10** Illustration of the intra-observer reproducibility for averaged Sinus of valsalva diameter measurements.


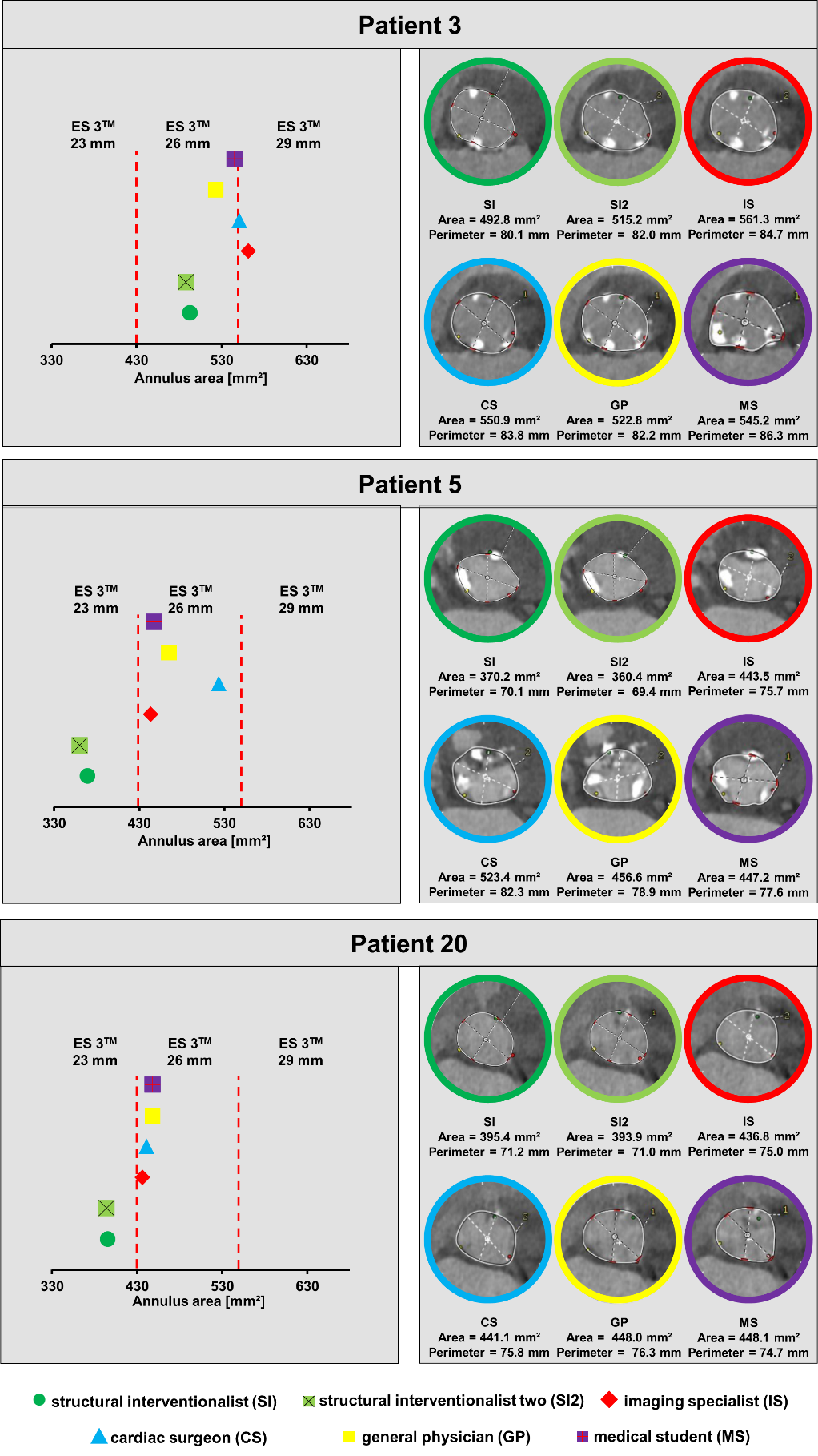


**Figure S 11:** Graphical illustration of the measured area in three different patients as well as the original MDCT images.

The dotted red lines in the plots represent valve size cut offs according to the recommendation sheets of the manufacturer.

In all cases the definition of the aortic annulus area varies between all observers as can be appreciated from the MDCT images.

Furthermore, while in patient 3 and 20 borderline measurements contributed to different valve sizing in patient 5 the delineation of the aortic annulus was furthermore particularly cumbersome because of the amount of calcification leading to difficulties in a clear border definition.
